# Cognitive training and promoting a healthy lifestyle for individuals with isolated REM sleep behavior disorder: study protocol of the delayed-start randomized controlled trial CogTrAiL-RBD

**DOI:** 10.1186/s13063-024-08265-9

**Published:** 2024-06-28

**Authors:** Anja Ophey, Sinah Röttgen, Julia Pauquet, Kim-Lara Weiß, Daniel Scharfenberg, Christopher E. J. Doppler, Aline Seger, Clint Hansen, Gereon R. Fink, Michael Sommerauer, Elke Kalbe

**Affiliations:** 1 Department of Medical Psychology | Neuropsychology and Gender Studies, Center for Neuropsychological Diagnostics and Intervention (CeNDI), University Hospital Cologne and Faculty of Medicine, University of Cologne, Cologne, Germany; 2 Cognitive Neuroscience, Institute for Neuroscience and Medicine (INM-3), Research Center Jülich, Jülich, Germany; 3 Department of Neurology, University Hospital Cologne and Faculty of Medicine, University of Cologne, Cologne, Germany; 4 Department of Neurology, University Medical Center Schleswig-Holstein, Campus Kiel, Kiel, Germany; 5 Center of Neurology, Department of Parkinson, Sleep and Movement Disorders, University of Bonn, Bonn, Germany

**Keywords:** Isolated REM sleep behavior disorder, Cognitive training, Randomized controlled trial

## Abstract

**Background:**

Isolated REM sleep behavior disorder (iRBD) is an early α-synucleinopathy often accompanied by incipient cognitive impairment. As executive dysfunctions predict earlier phenotypic conversion from iRBD to Parkinson’s disease and Lewy body dementia, cognitive training focusing on executive functions could have disease-modifying effects for individuals with iRBD.

**Methods:**

The study CogTrAiL-RBD investigates the short- and long-term effectiveness and the feasibility and underlying neural mechanisms of a cognitive training intervention for individuals with iRBD. The intervention consists of a 5-week digital cognitive training accompanied by a module promoting a healthy, active lifestyle. In this monocentric, single-blinded, delayed-start randomized controlled trial, the intervention’s effectiveness will be evaluated compared to an initially passive control group that receives the intervention in the second, open-label phase of the study. Eighty individuals with iRBD confirmed by polysomnography will be consecutively recruited from the continuously expanding iRBD cohort at the University Hospital Cologne. The evaluation will focus on cognition and additional neuropsychological and motor variables. Furthermore, the study will examine the feasibility of the intervention, effects on physical activity assessed by accelerometry, and interrogate the intervention’s neural effects using magnetic resonance imaging and polysomnography. Besides, a healthy, age-matched control group (HC) will be examined at the first assessment time point, enabling a cross-sectional comparison between individuals with iRBD and HC.

**Discussion:**

This study will provide insights into whether cognitive training and psychoeducation on a healthy, active lifestyle have short- and long-term (neuro-)protective effects for individuals with iRBD.

**Trial registration:**

The study was prospectively registered in the German Clinical Trial Register (DRKS00024898) on 2022–03-11, https://drks.de/search/de/trial/DRKS00024898. Protocol version: V5 2023–04-24.

**Supplementary Information:**

The online version contains supplementary material available at 10.1186/s13063-024-08265-9.

## Background

REM (rapid eye movement) sleep behavior disorder (RBD) is a parasomnia characterized by loss of muscle atonia and dream enactment during REM sleep [1, 2]. Isolated RBD (iRBD) is an early α-synucleinopathy [3–5] and may constitute a prodromal stage of Parkinson’s disease (PD), dementia with Lewy bodies (DLB), or multiple system atrophy (MSA) [6–10]. To date, iRBD is the most specific marker to indicate the prodromal stage of PD and a well-recognized feature in prodromal DLB and MSA [6–10]. Within 15 years after diagnosis, up to 80% of individuals with iRBD exhibit phenotypic conversion to PD, DLB, or MSA, with approximately 95% of the converters eventually developing PD or DLB [10, 11].

In the prodromal PD stage [12, 13], and particularly in individuals with iRBD [14, 15], cognitive alterations have been reported. As expected by definition, a recent multicenter data analysis by the International REM Sleep Behavior Disorder Study Group suggests that the only reliable clinical marker differentiating between individuals with iRBD who develop PD or DLB first is the higher prevalence and rate of cognitive decline in DLB-converters [11]. Furthermore, individuals with iRBD converting to PD are more likely to develop a more aggressive “diffuse malignant” PD subtype, characterized by a higher burden of non-motor symptoms, particularly cognitive dysfunction and eventually PD dementia (PDD) [11, 16–18]. Cognitive decline is one of the most debilitating non-motor symptoms in PD [19], as it greatly impacts the patients’ quality of life and independence in activities of daily living and increases the burden on caregivers, care providers, and the public healthcare system [20, 21].

Compared to healthy control individuals (HC), meta-analytical evidence suggests that individuals with iRBD show alterations of medium effect sizes in global cognition, executive functions, and memory [15]. Furthermore, impairments in global cognition and executive functions in individuals with iRBD have been identified as risk factors for conversion within the α-synucleinopathies [7, 14, 15]. Cognitive alterations in individuals with iRBD are accompanied by functional and structural alterations in the brain, pointing to early neurodegeneration at this stage [22–25]. Therefore, iRBD not only represents a clinical syndrome but may offer a window of opportunity for disease-modifying, potentially neuroprotective pharmacological and non-pharmacological interventions [10, 26, 27]. The development of neuroprotective interventions and their evaluation in clinical trials is of outstanding clinical interest. It could alleviate ethical concerns associated with an early iRBD diagnosis, as the latter informs individuals about their increased risk of developing PD, DLB, or MSA without available targeted interventions [27–29]. For Alzheimer’s disease, large (secondary) prevention trials already exist that focus on non-pharmacological multi-domain interventions in at-risk cohorts [30–32]. These interventions aim to promote a healthy, active lifestyle in the domains of cognition (e.g., through cognitive training), exercise (e.g., through physical exercise interventions), and nutrition (e.g., through information, consulting, and monitoring). These interventions have demonstrated long-lasting effects, particularly concerning cognitive functions [30, 32, 33].

Non-pharmacological interventions, especially cognitive interventions, have the potential to preserve cognitive functions in both healthy and pathological aging, thereby enhancing the quality of life and independence of older individuals. In a systematic review summarizing 46 meta-analyses on the effectiveness of cognitive interventions, Gavelin et al. [34] demonstrated that the existing evidence suggests reliable positive effects of cognitive training on global cognition of older adults. Meta-analyses have also shown positive training effects induced by cognitive training specifically for individuals with PD [35–37]. These effects were particularly evident in vulnerable cognitive domains in PD, such as executive functions [35–37]. This is particularly important as executive dysfunctions predict earlier conversion within α-synucleinopathies [7, 14, 15]. Therefore, cognitive training focusing on executive functions could have disease-modifying effects for individuals with iRBD. This hypothesis is supported by studies showing that cognitive training interventions cannot only improve cognitive functions at the behavioral level, but may also lead to network optimizations: Meta-analytically, reduced task-related activation in cortical areas along with increased task-related activation in subcortical areas was observed after cognitive training, which is interpreted as an increase in neural efficiency [38, 39]. Aside from the growing evidence for overall beneficial behavioral and neural effects of cognitive training, the individual responsiveness to cognitive interventions may be heterogeneous. Thus, only an in-depth characterization of the participants’ baseline prerequisites, e.g., in terms of demographic, clinical, (neuro-)psychological, or structural and functional brain parameters, may foster the understanding of who benefits most from cognitive interventions and help to tailor interventions individually [40–43].

Individuals with iRBD and individuals with PD plus RBD often exhibit severe degeneration of the noradrenergic locus coeruleus, which is an established predictor of cognitive decline in PD [44, 45]; however, the relevance of the noradrenergic system in the context of cognitive training is unknown. Given that behavioral effects of cognitive training can endure up to 10 years in healthy older individuals [33], it is unsurprising that this kind of intervention might trigger not only functional but also structural brain changes (e.g., gray matter and cortical volume increases) [46]. However, no data on the plasticity of white and gray matter microstructure upon a cognitive intervention in iRBD is available, and data in PD remains scarce and inconclusive [47, 48]. More studies elucidating the mechanisms of neural plasticity induced by cognitive interventions via longitudinal imaging before and after cognitive training are warranted, particularly for individuals with iRBD.

A plethora of data suggests a mutual relationship between sleep and cognition in health and disease [49], and the deterioration of sleep macro- and microparameters in individuals with PD has been demonstrated repeatedly [1, 50, 51]. Furthermore, individuals with PD with a lower amount of slow-wave sleep are prone to a faster motor progression as well as poorer cognitive performance [52]. Hence, sleep macro- and microparameters may constitute a relevant driver of the responsiveness to cognitive interventions in iRBD, and data on sleep plasticity are warranted to guide future interventional trials.

The study CogTrAiL-RBD (Cognitive Training and a Healthy, Active Lifestyle Program for Patients with isolated REM Sleep Behavior Disorder) aims to investigate the short- and long-term effectiveness and the feasibility of a cognitive training intervention for individuals with iRBD. The intervention consists of (i) a 5-week digital cognitive training and (ii) a module promoting a healthy, active lifestyle, which includes psychoeducational information on cognitive, physical, and social lifestyle activities, as well as nutrition, along with concurrent monitoring of these domains using a digital activity diary over a period of 15 months.

Within the framework of a monocentric, single-blinded, delayed-start randomized controlled trial, the intervention’s effectiveness will be evaluated compared to an initially passive control group that receives the intervention in the second, open-label phase of the study. The evaluation will focus on cognition and additional neuropsychological and motor variables. Furthermore, the general feasibility of the intervention for individuals with iRBD will be investigated. Additionally, the study will examine the neural effects of the intervention by magnetic resonance imaging (MRI) and overnight polysomnography. A healthy, age-matched control group will be examined at the first assessment time point, enabling a cross-sectional comparison between the individuals with iRBD and HC. Reporting of this study protocol follows the “Standard Protocol Items: Recommendations for Interventional Trials” (SPIRIT) checklist [53]. A schematic figure of the study design can be found in Appendix Figure A-1.

## Methods/design

### Study design and setting

The study CogTrAiL-RBD is designed as a monocentric, single-blinded, delayed-start randomized controlled trial with two study arms aiming to consecutively recruit 80 individuals with iRBD confirmed by polysomnography from the continuously expanding local iRBD cohort at the University Hospital Cologne in Germany [54]. Participants will be randomly assigned to either the early intervention group or a delayed-start control group. Forty participants will be randomly assigned to each group (parallel group phase, superiority). The delayed-start control group will receive the same intervention after a 6-month waiting period, with the early intervention group undergoing the intervention for a second time to separate symptomatic from potentially disease-modifying effects (open-label phase, exploratory) [55].

Figure A-1 presents the schematic trial design and Appendix Figure A-1 further illustrates the timeline for participants. At baseline assessment (t0, week 0), the study will be explained to the participants. Subsequently, the participants will provide written informed consent and answer to a demographic interview. All participants with iRBD will undergo comprehensive clinical and neuropsychological assessments at the t0 visit and 6 weeks post allocation (posttest I, t1, week 6). A follow-up assessment (follow-up I, t2, week 33) will be conducted after 6 months. Subsequently, the second intervention phase for both the early intervention group and the delayed-start control group begins, followed by another clinical and neuropsychological assessment (posttest II, t3, week 39). Finally, a follow-up assessment (follow-up II, t4, week 66) will take place after an additional 6 months.

There are three optional study modules for individuals with iRBD that accompany the mandatory clinical and neuropsychological assessments: (i) the MRI module consisting of structural and functional MRI at each assessment time point; (ii) the accelerometry module consisting of physical activity tracking with accelerometers at each assessment time point; and (iii) the polysomnography module including two polysomnographies, one before the start of the intervention and one within the last 2 weeks of the intervention. Additionally, an age- and sex-matched HC group will be examined at the first assessment time point, participating in the comprehensive clinical and neuropsychological assessments, the MRI module and the accelerometry module.

### Recruitment

Individuals with iRBD are recruited from the local iRBD cohort at the University Hospital Cologne, Germany, which is continuously expanding through active recruitment efforts. The recruitment strategy for the local iRBD cohort has been described in Seger et al. [54]. Those who consented to be contacted for study participation during the annual iRBD consultation hours are directly approached by the CogTrAiL-RBD study personnel via telephone. The subjects are provided with a comprehensive information sheet outlining the details of CogTrAiL-RBD, and, if interested in study participation, a structured telephone interview is conducted. The HC group will be recruited via email newsletters, flyers, and newspaper advertisements.

### Inclusion and exclusion criteria

The following inclusion criteria are applied for individuals with iRBD: (i) polysomnography-proven diagnosis of iRBD, (ii) age between 40 and 80 years, (iii) normal or corrected-to-normal vision and hearing, (iv) German as native tongue or sufficient proficiency in German, and (v) access to a local computer with access to the internet. Exclusion criteria for individuals with iRBD are (i) severe cognitive dysfunctions (Montréal Cognitive Assessment, MoCA, ≤ 22) [56] interfering with the ability to give informed consent, (ii) significant neurological and psychiatric concomitant diseases, and for those willing to participate in the optional MRI module (iii) contraindications for MRI. Except for the polysomnography-proven diagnosis of iRBD, the same in- and exclusion criteria are applied for HC plus the absence of a diagnosis of a movement disorder or signs of iRBD or any other psychiatric and neurological condition as assessed by medical history.

### Sample size and power calculation

To determine an adequate sample size, an a priori power analysis was conducted with G*Power [57] (http://www.gpower.hhu.de). The study will be powered to reliably detect behavioral effects in the domain of executive functions (the primary outcome) in the first parallel-group phase of the randomized controlled trial in individuals with iRBD. According to two meta-analyses on cognitive training in healthy older adults [58] and individuals with PD [36], we expect a medium effect size (Cohen’s *d* = 0.5) in the domain of executive functions. With a two-sided *α*-level of 0.05 and 80% power, the minimum sample size comparing the difference between two independent groups (allocation ratio 1:1) with a *t*-test is *N* = 128 (i.e., *n* = 64 per group).

We applied the correction formula of Borm et al. [59], acknowledging the increase of precision in estimating a treatment effect when the correlation between the outcome measure at baseline and retest ($\rho$) by ANCOVA is considered: *n*_cor_ = n*$\sqrt{1-{\rho }^{2}}$. A meta-analysis [60] on the reliability of neuropsychological measures identified particularly high correlations between baseline and retest measures of executive functions (Pearson’s *r* ≥ 0.80). Following, the G*Power estimate of *n* = 64 per group was corrected by the factor $\sqrt{1-{0.8}^{2}}=0.6$, resulting in a minimum sample size of *n*_cor_ = 38 per group, i.e., a total sample of *N*_cor_ = 76. The estimated dropout rate for the first parallel-group phase of the randomized controlled trial of the present trial is 5%, based on previous experiences with a similar intervention in individuals with PD [61]. Following, we aim to recruit *N* = 80 (= 76/0.95) individuals with iRBD for the present trial.

### Patient adherence

Following recent recommendations for enhancing participant engagement in clinical studies [62], every participant has one contact person throughout the study. Every participant is welcomed by one of the principal investigators at the t0 visit, and this individual contact person is present at every following study visit, if possible. All participants will receive an information folder containing an overview of the study, contact information, and a schedule of appointments, where they can store all study documents (such as study information, insurance information, and consent forms). The participants will be reminded of their following appointments by email 1 week before the scheduled date. At every appointment and during each contact, the participant’s individual contact person will actively seek out any open questions and remarks and will provide assistance accordingly. At the end of the study, participants will receive individual feedback about their neuropsychological test performance, if desired, and a travel cost subsidy (max. € 150 for individuals with iRBD, € 60 for HC individuals). If, for whatever reason, complete study adherence is not possible, an effort will be made to collect as much data as possible from the respective participant.

### Randomization and blinding

Randomization in CogTrAiL-RBD is based on a minimization procedure designed to minimize differences between the early intervention group and the delayed-start control group in age, sex, and premorbid IQ [63]. The minimization randomization is implemented using the R package *Minirand* [64]. We aim at a 1:1 ratio of the number of subjects in the early intervention group and the delayed-start control group. The covariates age, sex, and premorbid IQ will be equally weighted (1/3 each). The participants with iRBD will be consecutively randomized following the clinical and neuropsychological t0 baseline assessment by a person not involved in t1 to t4 data collection, preferably by their individual contact person, who will assign the participants to the intervention and monitor their training progress.

The assessments will be double-blinded at t0 baseline (i.e., randomization will be carried out after the t0 baseline assessment) and single-blinded at t1 to t4 posttests and follow-ups (i.e., outcome assessors do not know the participant’s group allocation). Any violations of blinding will be documented, e.g., if participants do not fully comply with the explicit instruction not to give any clues about their experimental group allocation to the outcome assessors.

### Cognitive training intervention

The cognitive training intervention consists of a 5-week digital cognitive training and a module promoting a healthy, active lifestyle including psychoeducational information on a healthy, active lifestyle and activity monitoring using a digital activity diary.

#### Computerized multi-domain cognitive training

The computerized training will be delivered by the CE-certified class-I medical device “HeadApp/NEUROvitalis Digital” (HelferApp GmbH, https://start-headapp.helferservices.net) offering adaptive, multi-domain cognitive training accessible from home with internet access. Participants will train for a total of 15 training sessions across 5 weeks, 40 min per day, three times a week resulting in a total training duration of 600 min, which is comparable to previously published interventions [34]. The training consists of six different tasks addressing one primary cognitive domain each: executive functions, working memory, episodic memory, attention, visuo-cognition, and language. The training starts with the lowest difficulty and adapts to training performance concerning higher difficulty and task complexity levels. With higher difficulty levels, all tasks involve a strong executive component, leading to a strong executive focus of the applied cognitive training. The training tasks are based on the scientifically validated NEUROvitalis program (ProLog, Therapie und Lernmittel GmbH, Cologne) [65–69]. Depending on their group assignment, participants receive their login credentials and information about the training during their t0 or t2 visit. The training will be monitored weekly on the HeadApp/NEUROvitalis Digital website by the individual’s contact person, and any queries of participants will be answered by telephone or email, depending on the participant’s choice.

#### Healthy lifestyle information

The intervention is enriched by psychoeducation promoting a healthy, active lifestyle. Topics include risk and protective factors of healthy aging, cognitive and motor reserve, advice for incorporating more cognitive, physical, and social lifestyle activities into daily life routines, the influence of diet on (cognitive) health, and information on the Mediterranean diet. A graphic booklet will deliver the healthy lifestyle information to the participants at the beginning of the intervention period, i.e., at the t0 or the t2 visit. The psychoeducation booklet is based on the scientifically validated NEUROvitalis program (ProLog, Therapie und Lernmittel GmbH, Cologne) [65–69].

#### Weekly/monthly activity diary

During the study, participants of the early intervention group will fill out weekly (between t0 and t1, t2 and t3) and monthly (between t1 and t2, t3 and t4) digital activity diaries, reporting their cognitive and social lifestyle activities, physical activity level, and Mediterranean diet adherence. Participants of the delayed-start control group will fill out the weekly and monthly digital activity diaries following their introduction to the training following the t2 visit. With this monitoring, we aim at a continuous reflection of activity levels in the domains of the psychoeducational program of participants, motivating the implementation of new active daily life routines. To assess these lifestyle activities, we use established, validated questionnaires. The short version of the International Physical Activities Questionnaire (IPAQ) [70, 71] is used to assess physical activity levels, cognitive and social lifestyle activities are assessed with the Lifestyle Activities Questionnaire (LAQ) [72, 73], and adherence to the Mediterranean diet with the Mediterranean Diet Adherence Screener (MEDAS) [74, 75]. In the weekly version of the activity diary, the questions refer to the past week and in the monthly version they are related to the past month. For each lifestyle domain (cognitive and social lifestyle activities, physical activity, and Mediterranean diet adherence), we additionally ask for subjective ratings of the participant’s activity level in general and compared to the past week/the past month on a 4-point Likert scale. The activity diary questionnaires are sent out by email via SoSci Survey (SoSci Survey GmbH, Munich, Germany). Mailing lists are generated in *R* and directly uploaded to SoSci Survey to automatically send out individualized links to fill out the activity diaries at the planned time points.

### Assessments

An equally weighted domain composite score of executive functions will constitute the primary outcome of the present study. The other cognitive domain (visuo-cognition, attention and working memory, memory, language) equally weighted composite scores, an equally weighted global cognition composite score based on the equally weighted domain composite scores, single test scores, as well as the assessments of non-motor symptoms, (fine) motor abilities, quality of life, and the feasibility of the intervention constitute the secondary outcomes. Physical activity measured by accelerometry, brain imaging parameters, and polysomnography parameters constitute exploratory outcomes.

#### Neuropsychology and motor assessments

All subjects will undergo clinical and neuropsychological assessments, including paper–pencil cognitive and motor evaluations. The in-person assessments take around 2.5 h to complete, including a short break according to the participant’s needs. The test battery will be administered by psychologists or graduate students of Psychology or Medicine at all time points. The outcome assessors will be thoroughly trained in administering and scoring the assessments. Following the in-person assessments, participants digitally fill out questionnaires on various non-motor symptoms, quality of life, and lifestyle activities, which are sent to them via SoSci Survey (SoSci Survey GmbH, Munich, Germany). An automatic reminder email is sent to the participants, if they do not complete the questionnaire within 1 week of their study visit. A complete list of all cognitive assessments, motor evaluations, and questionnaires, including references, is presented in Table 1. Parallel test forms are used if available (i.e., alternating between time points). Test scores are standardized into *z*-scores, percentage ranks (PR), or *T*-scores using published normative data, as available. All evaluations are double-checked and digitalized using the four-eye principle. All standardized scores are transformed into *z*-scores during data preprocessing to create the domain composite scores. Table 1 provides an overview of the assignment of cognitive tests to the respective domains.

**Table 1:** Neuropsychological and motor assessments in CogTrAiL-RBD

Domain	Abbreviation	Assessment	Reference	Baseline	Posttest I and II	Follow-up I and II
**Cognition**						
Cognitive reserve	LEQ	Lifetime of Experiences Questionnaire	Valenzuela and Sachdev [76]; Roeske et al. [77]	X		
Cognitive testing				X	X	X
**Overall cognitive state**						
Subjective cognition	Multi-SubCoDE	Multiple Domain Subjective Cognitive Decline Evaluation	Not published yet, available upon request from the corresponding author			
Global cognition	MoCA	Montreal Cognitive Assessment: Version B or A	Nasreddine et al. [56]			
Social cognition	RMET	Reading the Mind in the Eyes Test	Baron‐Cohen et al. [78], Kynast et al. [79]			
**Executive**						
Semantic fluency	RWT sem	Regensburger Wortflüssigkeitstest: Food or Animals	Aschenbrenner et al. [80]			
Phonemic fluency	RWT phon	Regensburger Wortflüssigkeitstest: P- or S-words	Aschenbrenner et al. [80]			
Set-shifting	TMTB/A	Trail Making Test (TMT): TMT-B/TMT-A	Reitan [81]; Aebi [82]			
Inhibition	Stroop-I	Stroop Interference	Bäumler and Stroop [83]			
Logical reasoning	LPS-4	Leistungsprüfsystem 50 + : Subtest 4, Fluid Reasoning, Version A or B	Sturm et al. [84]			
**Visuo-cognition**						
Construction	ROCFT	Rey Osterrieth Complex Figure Test (ROCFT): Figure Copy	Rey [85], Strauss et al. [86]			
Perception	LPS-11	Leistungsprüfsystem 50 + : Subtest 11, Visual Perception, Version A or B	Sturm et al. [84]			
Spatial perception	BJLO	Benton Judgment of Line Orientation, Version V or H	Benton et al. [87], Benton [88]			
	LPS-7	Leistungsprüfsystem 50 + : Subtest 7, Spatial Rotation, Version A or B	Sturm et al. [84]			
**Attention and working memory**						
Working memory	DSback	Wechsler Adult Intelligence Scale (WAIS): Digit Span backwards	Wechsler [89]			
	BTA	Brief Test of Attention	Schretlen [90]			
Processing speed	TMT-A	Trail Making Test A	Reitan [81]; Aebi [82]			
Attention	Stroop-W	Stroop Word	Bäumler and Stroop [83]			
	Stroop-C	Stroop Color	Bäumler and Stroop [83]			
**Memory**						
Verbal memory	DSforw	Wechsler Adult Intelligence Scale (WAIS): Digit Span forwards	Wechsler [89]			
	VLMT-Learn	Verbaler Lern- und Merkfähigkeitstest (VLMT): Wordlist Learning, Version C or A	Helmstaedter and Durwen [91]			
	VLMT-Rec	Verbaler Lern- und Merkfähigkeitstest (VLMT): Wordlist Recall, Version C or A	Helmstaedter and Durwen [91]			
Visuo-spatial memory	ROCFT	Rey Osterrieth Complex Figure Test (ROCFT): Figure Recall	Rey [85], Strauss et al. [86]			
**Language**						
Naming	ACL-Naming	Aphasia Check List, Subtest Naming	Kalbe et al. [92]			
Semantic and abstraction	WAIS	Wechsler Adult Intelligence Scale (WAIS): Similarities	von Aster and Neubauer [93]			
**Non-motor**						
Depressive symptoms	BDI-II	Beck Depression Inventory (BDI-II)	Beck et al. [94]	X	X	X
Fatigue	FSMCF	Fatigue Scale for Motor and Cognitive Functions	Penner et al. [95]	X	X	X
Quality of life	SF-36	Short Form Health 36 (SF-36 V2.0)	Ware et al. [96]; Bullinger [97]	X	X	X
Perception of stress	PSQ	Perceived Stress Questionnaire	Levenstein et al. [98]; Fliege et al. [99]	X	X	X
Self-efficacy	SWE	Skala zur Allgemeinen Selbstwirksamkeitserwartung	Jerusalem and Schwarzer [100]	X		
Non-motor symptoms	NMSQ	Non-Motor Symptoms Questionnaire (NMSQ)	Chaudhuri et al. [101]	X	X	X
Lifestyle activities	LAQ	Lifestyle Activities Questionnaire	Carlson et al. [72], Parisi et al. [73]	X	X	X
Diet	MEDAS	Mediterranean Diet Adherence Screener	Schröder et al. [74], Hebestreit et al. [75]	X	X	X
**Motor**						
Motor reserve	PASE	Physical Activity Scale for the Elderly (PASE)	Washburn et al. [102]	X		
Physical activity	IPAQ	International Physical Activity Questionnaire (IPAQ)	Craig et al. [70], Lee et al. [71]	X	X	X
PD motor symptoms	MDS-UPDRS-III	Movement Disorder Society Unified Parkinson’s Disease Rating Scale Part III	Goetz et al. [103]	X	X	X
Dual tasking	TuG	Timed Up and Go Test—Single and Dual Task Condition	Podsiadlo and Richardson [104], Zirek et al. [105]	X	X	X
Fine motor skills	PPT	Purdue Pegboard Test	Tiffin and Asher [106], Agnew et al. [107]	X	X	X
**Sleep**						
Subjective sleep quality	PDSS	Parkinson’s Disease Sleep Scale	Trenkwalder et al. [108]	X	X	X
RBD symptom severity	RBDSQ	REM sleep behavior disorder screening questionnaire	Stiasny‐Kolster et al. [109]	X		
	RBD-I	Innsbruck REM sleep behavior disorder inventory	Frauscher et al. [110]	X		

The motor examination with the motor part of the Movement Disorder Society (MDS) Unified Parkinson’s Disease Rating Scale (MDS-UPDRS-III) will be conducted and videotaped by outcome assessors and rated by one of two movement disorder specialists blinded for the diagnostic group (HC vs. RBD), experimental group allocation (early intervention group vs. delayed-start group) and time point of assessment (t0, t1, t2, t3, t4). The same movement disorder specialist will rate all videos of one subject. Ten randomly selected videos of the first 100 MDS-UPDRS-III recordings were double-rated by the two movement disorder specialists and the MDS-UPDRS-III total score reached an intraclass correlation (ICC) of 0.87, indicating good to excellent interrater reliability. Rigidity assessments as part of the UPDRS-III are conducted and rated during the test sessions by the outcome assessors. All outcome assessors are previously trained by the movement disorder specialists.

#### Feasibility

As this clinical trial constitutes one of the first non-pharmacological intervention studies for individuals with iRBD, the feasibility assessment of such an intervention is of great interest. We will report the participation score, i.e., the proportion of individuals of the continuously expanding Cologne iRBD cohort willing to participate in the intervention study (regardless of subsequent randomization). Additionally, within the intervention study, factors of interest include adherence to the intervention protocol (compliance) in terms of the rate of successful completion of the cognitive training protocol operationalized as the completion of at least 80% of planned training sessions, equivalent to a minimum of 12 training sessions, and the consistent utilization of the digital activity diary. Furthermore, the overall dropout rate across data collection time points will be compared between the early intervention group and the delayed-start control group. While the study does not incorporate a systematic collection of qualitative feedback, participants are encouraged to provide feedback on the cognitive training and activity diary and communicate any technical challenges they encounter to their designated contact person throughout the study.

#### Accelerometry: physical activity tracking

All willing participants will be asked to wear a wrist-mounted movement sensor measuring linear acceleration and angular velocity on their dominant hand for 7 days around each assessment time point. The sensors will be distributed either during the assessment session (along with a prepaid return envelope) or sent to the participants 10 days prior the next assessment. The participants will be instructed to wear the sensor as much as possible throughout their days and nights. Additionally, participants will be asked to fill out a diary noting their bedtimes, daily activities, and periods when they are not wearing the device.

The CE-certified movement sensor used in this study is the AX6 from Axivity Ltd, Newcastle upon Tyne, England. Detailed information about the AX6 and the wrist strap can be found in the Data Sheet documents available on the Axivity website (https://axivity.com/product/ax6). The sensors will be configured to record data continuously for 7 consecutive days and nights (168 h) at a sampling rate of 100 Hz with an accelerometer range of ± 8 g. Sensor set-up and data storage will be managed using the open source AX3/AX6 OMGUI Configuration and Analysis Tool (https://github.com/digitalinteraction/openmovement/wiki/AX3-GUI). Data retrieval from the sensor is only possible via a USB cable connection. Data will be saved in the Continuous Wave Accelerometer (*.cwa) binary format containing the raw actigraphy data, metadata, and device configurations.

#### Magnetic resonance imaging

Extensive brain imaging with a 3 T SIEMENS PRISMA scanner equipped with a 64-channel head coil will be conducted at the Research Center Jülich near Cologne for all willing participants at all time points (t0–t4). The MRI scanning will preferably occur within 7 days of the clinical and neuropsychological assessment. However, in case of technical difficulties with the scanner or timing issues with the participants (e.g., illness or personal reasons), it may be conducted up to 3 weeks apart. During MRI measurements, at least one member of the study team is present. This person will be supported either by another member of the study team or by a medical-technical radiology assistant of the Research Center Jülich. The net scanning time for all sequences lasts 69:33 min. The sequences, their features and specifications, spatial resolution, repetition times (TR), and echo times (TE) as the basic pulse sequence parameters are described in Table 2. The MRI data will be exported and saved in the DICOM format (*.dcm).

**Table 2:** Magnetic resonance imaging protocol CogTrAiL-RBD

Sequence	Features	Duration
T1-weighted	The T1-weighted sequence will be used to monitor cortical thickness, gray matter volume, and deformation. These metrics are well-established markers which might change upon training. Sequence parameters: 0.9 mm isotropic MP2RAGE, TR 2500.0 ms, TE 2.22 ms	6:24 min
Neuromelanin-sensitive turbo spin echo	Turbo spin-echo (TSE) sequences allow for contrast evaluation of neuromelanin containing structures, i.e., locus coeruleus and substantia nigra. Measures of locus coeruleus integrity like volume, voxels with highest intensity as well as contrasts of spatial subdivisions will be performed [111]. Such measures will elucidate the role of an intact locus coeruleus as prerequisite for successful cognitive training. Sequence parameters: 0.4 × 0.4 × 1.8 mm^3^ resolution, TR 825.0 ms, TE 18.00 ms	7 × 2:24 min = 16:48 min
Multi-shell diffusion	We will analyze metrics of white and gray matter microstructure obtained via modeling of the measured multi-shell diffusion-weighted signal, including diffusion kurtosis imaging (DKI), neurite orientation dispersion and density imaging (NODDI), and free-water mapping [112]. Multi-shell diffusion additionally opens the possibility for advanced fiber tracking approaches allowing to disentangle complex fiber configurations (e.g., crossing fibers) [113]. These microstructural data were shown to correlate with motor and cognitive performance in PD and might be particularly sensitive to plastic changes of brain organization. Sequence parameters: 1.5 mm isotropic resolution, TR 3800.0 ms, TE 90.00 ms	6:32 min + 0:50 min + 0:50 min + 6:36 min + 4:07 min = 18:48 min
Task-based functional MRI	The Wisconsin Card Sorting Task will be applied during functional MRI (fMRI) as an established test engaging executive functions and working memory. We chose an established task including multiple cognitive domains to study broad effects of functional reorganization upon cognitive training. Due to the longitudinal study design, we will be able to monitor transitions of the activation pattern correlating to training effects and baseline cognitive capacity. To implement the Wisconsin Card Sorting Test (WCST) in the present study, the adapted version of the WCST [114] was further adapted for functional MRI measurements according to Lie et al. [115]. The WCST is programmed in Presentation®, Version 23, of NeuroBehavioral System Inc. Changes compared to the standard WCST include a fixed response time, which is necessary to create meaningful blocks for MRI analysis and ensure the same task duration for all participants. For presentation in the scanner using a mirror, the task is programmed in a mirror-reversed manner. Before conducting the task in the scanner, participants are asked to practice a brief version of the task on the experimenter’s computer to clarify any questions. The experiment randomizes both the order of target categories and the order of cards for each participant. Responses are made using key presses on the computer during the practice session and using a hand response device (Celeritas) in the scanner. To perform the task in the scanner, the keyboards need to be tested, and full visibility of the monitor through the mirror must be ensured. Sequence parameters: 2.0 mm isotropic resolution, TR 800.0 ms, TE 37.00 ms	12:28 min
Resting-state functional MRI	Resting-state fMRI is a highly established imaging modality to study functional connectivity of the brain. We will apply more advanced measures of dynamic functional connectivity, which we recently applied in patients with PD [116]. Sequence parameters: 2.0 mm isotropic resolution, TR 800.0 ms, TE 37.00 ms	10:20 min
Quantitative susceptibility mapping	Quantitative susceptibility mapping (QSM) measures cerebral iron content. QSM is susceptible for tissue changes in nigrostriatal dopaminergic pathways [117]. Sequence parameters: 0.9 mm isotropic resolution, TR 47.0 ms, TE1 7.38 ms, TE2 14.76 ms, TE3 22.14 ms, TE4 29.52 ms, TE5 35.76 ms, TE6 42.00 ms	4:45 min
	Total net scanning time	69:33 min

*MRI* magnetic resonance imaging, *TE* echo time, *TR* repetition time

#### Polysomnography

All participants with iRBD undergoing MRI will also be invited to participate in two polysomnography sessions during their (first) period of cognitive training. For participants in the early intervention group, the polysomnography sessions will take place between t0 and t1, while for participants in the delayed-start control group, the sessions will occur between t2 and t3. The first polysomnography session will be conducted before the start of cognitive training, and the second session will be held in the last or second-to-last week of training, following a training day. The participants’ sleep prior to and during cognitive training will be monitored using mobile polysomnography equipment including 10 EEG channels, electromyography (EMG) of the chin, arms, and legs, surveillance of breathing efforts, as well as high-definition infrared (IR) videorecording. The SOMNOscreen plus device from SOMNOMedics GmbH, located in Randersacker, Germany, will be utilized.

The mobile nature of the polysomnography device allows for examinations to be conducted in the participants’ homes or hotel rooms, providing high convenience for the subjects. All participants are already familiar with the polysomnography examination from their iRBD diagnostic process, minimizing first-night effects. The polysomnography set-up takes approximately 90 min. After completing the set-up, participants can freely move as there are no cables restricting movement and they can spend their evening to their liking and go to bed at their preferred time. The following morning, a member of the study team will detach the polysomnography equipment. The polysomnography data will be saved in the proprietary file format of SOMNOMedics GmbH, which can be converted to the European Data Format EDF + format (*.edf), which is commonly used to store multichannel medical time series data.

### Data management and monitoring

All collected data will be pseudonymized with an individual identification number following the format RBD-XXX for individuals with RBD and HC-RBD-XXX for HC individuals, where XXX represents a non-consecutive, randomly generated three-digit number. Paper-based data such as the scoring sheets of the clinical and neuropsychological assessments will be stored in lockable cabinets in a room with restricted access at the University Hospital Cologne, sorted by participant ID for easy access at each stage of the study. Data acquired on paper will be digitalized by one staff member and double-checked by another. The progress of data collection, scoring, digitalization, and monitoring will be documented. Prior to data analysis, range checks for data values will be conducted. All digitally acquired data, such as questionnaire data from SoSci Survey (SoSci Survey GmbH, Munich, Germany), the MDS-UPDRS-III videos, the accelerometry module (*.cwa files), and the PSG module (proprietary file format and *.edf files), will be saved on a secure server of the University Hospital Cologne with restricted access only to the staff involved in this project. The MRI data (*.dcm files) is sent to a Picture Archiving and Communication System (PACS) and stored in the study archive of the Research Center Jülich in a data protection-compliant and pseudonymized manner. Spreadsheets concerning sensitive data, such as the name-ID key list, names, and contact information, will be further protected by a password and saved on a secure server with access only to the staff involved in participant management of the study. Additionally, all digitally acquired data will be backed-up on an encrypted hard drive stored in a lockable cabinet in a room with restricted access at the University Hospital Cologne. Following good scientific practice, data will be stored for at least 10 years. The final data set can be accessed by the study contributors at the University Hospital Cologne and the Research Center Jülich. Participants will also be requested to authorize the research team to share the pseudonymized data with members of other research groups from university and non-university research institutions for in-depth data analysis within non-commercial research projects. This study does not involve the collection or storage of biological samples. Auditing is done on a monthly basis for internal reasons, a yearly report is sent to the sponsor.

### Expected harms and adverse events monitoring

During the execution of clinical and neuropsychological assessments, accelerometry, cognitive training, and the healthy, active lifestyle module, we expect no risks or complications and subsequent issues, except for mild transient fatigue. The MRI is a non-invasive procedure for functional and structural imaging of tissues, utilizing strong magnetic fields and high-frequency radio waves without involving radioactively labeled ligands or X-rays. To minimize the risk of any MRI-related complications, the detailed medical history of participants is assessed to determine their MRI eligibility. Prior to scanning, participants are asked to remove all metallic objects and empty their pockets. Under careful consideration of inclusion and exclusion criteria, no known health risk exists for patients undergoing MRI. Experienced specialist physicians in (neuro-)radiology evaluate every MRI scan diagnostically about incidental findings. If any need for further medical measures arises from the review of the MRI examination, the study physician will inform and support the participants accordingly upon their request. All participants are informed about the possibility of incidental findings and their medical, legal, insurance-related, and psychological consequences. The burden on the participants from the mobile polysomnography is considered low since the individuals with iRBD are familiar with the procedure from the diagnostic process. The surface electrodes are applied similarly to adhesive patches and participants can sleep in their familiar environment or a similar setting (e.g., a hotel room). Some participants may report a mild to moderate sensation of foreign objects.

Overall, under careful consideration of the inclusion and exclusion criteria, the risks of the study are minimal. Participants will be informed about possible risks and adverse events (AE) at their t0 visit. Participants can withdraw from the study at any point without providing a reason and without facing any disadvantages. No written explanation is required, and a verbal statement is sufficient for dropout. Outcome assessors and study coordinators will be instructed to monitor and document all AE throughout the study. If a serious AE occurs, the study physician will be consulted and asked to assess whether or not a causal relationship with the intervention is considered possible. Beyond the trial, participants will be part of the local iRBD cohort, receiving access to approximately yearly clinical visits and additional visits as needed.

### Statistical analyses

In the present study protocol, we will report the statistical analyses for the primary and secondary outcomes of the neuropsychological and motor assessments in the first parallel-group phase of the randomized controlled trial. Separate analysis plans for the exploratory outcomes physical activity measured by accelerometry, brain imaging parameters, and polysomnography and exploratory analyses (e.g., subgroups) will be preregistered in the Open Science Framework (OSF, https://osf.io/). The statistical analyses will be conducted after the last t2 visit of the last participant. Data preprocessing, statistical analyses, and data visualization will be conducted in R [118] with commonly used statistical libraries, e.g., dplyr, lme4, and ggplot2.

For the baseline comparisons between the early intervention group and the delayed-start control group, independent sample *t*-tests, Wilcoxon rank-sum tests, or χ^2^ tests will used as appropriate. Variables and test scores will be previously inspected for normal distribution by Shapiro–Wilk tests. As effect sizes, Cohen’s *d* will be reported for *t*-tests and *r* for Wilcoxon rank-sum tests, indicating small (*d* ≥ 0.2; *r* ≥ 0.1), moderate (*d* ≥ 0.5; *r* ≥ 0.3), or strong (*d* ≥ 0.8; *r* ≥ 0.5) effects.

For the analysis of training effects in the primary and secondary outcomes, linear mixed effects (LME) models will be used. Dependent variables are the respective primary or secondary outcome measures. All dependent variables are assessed at three points of time. The LME models include time (t0, t1, t2), group (early intervention group and delayed-start control group), and the interaction between time and group (time*group) as fixed factors. Furthermore, participants and time are included as random factors. Models will be estimated using the *lmer()* function of the *lme4*-package employing restricted maximum likelihood estimation [119]. Model fit will be evaluated using marginal *R*^2^, which considers the variance of the fixed effects only, and conditional *R*^2^, which accounts for both fixed and random effects. *t*-Tests will be conducted to assess the significance of single coefficients. For the interaction effects, relative effect sizes with 95% confidence intervals will be reported and defined as the difference of the mean change over time in the early intervention group minus mean change over time in the delayed-start control group divided by the pooled baseline standard deviation of the cohort. Within the analysis of training effects, we will pursue an intention-to-treat approach. No imputation methods will be used, as one strength of LME models is their ability to deal with unbalanced designs, for example, due to missing values in longitudinal data.

### Dissemination plans

The results of CogTrAiL-RBD will be published in international, peer-reviewed scientific journals and presented at national and international conferences. Following the main publication of the behavioral effects of the training including the training effect on the primary outcome of executive functions, results will be disseminated to participants via email in a way that is easily understandable for non-experts. If any important protocol modifications occur, they will be communicated in a written form to relevant parties.

## Discussion

CogTrAiL-RBD is to the best of our knowledge the first cognitive intervention study, additionally promoting a healthy, active lifestyle, for individuals with iRBD. With the longitudinal, multi-modal data collection, it will not only be possible to gain insights on the effectiveness of such an intervention at this at-risk group for the development of PD, DLB, and MSA and corresponding cognitive impairment, but we will also develop a better understanding on the underlying mechanisms of behavioral training effects. The trial is powered to detect behavioral differences in the first parallel-group phase of the randomized controlled trial. Following, any analyses focusing on the second open-label phase are fully exploratory, constituting a limitation to the trial. From a clinicians’ and patients’ perspective, this trial is pioneering, as it could alleviate ethical concerns associated with an early iRBD diagnosis [27–29]. Cognitive training combined with psychoeducation on a healthy, active lifestyle would be a low-threshold and safe intervention approach for this at-risk group to counteract cognitive decline.

## Trial status

The trial status is ongoing with the first participant included on June 14, 2022. We expect the recruitment to be finished by June 2024. At the time of revision (May 10, 2024), 85% (*n* = 68) of the participants with iRBD planned to be included (*N* = 80) were recruited, with 68.75% (*n* = 55) already being enrolled in the trial. At baseline t0, the current participation score in the MRI module is 54.5% (*n* = 30), of which 56.7% (*n* = 17) were also willing to participate in the polysomnography module. The participation score for the accelerometry module is as high as 96.4% (*n* = 53) at baseline t0. The protocol version is V5 as of 2023–04-24.

### Supplementary Information

Additional file 1: Appendix Figure A-1 and Appendix B-2.

Additional file 2: Fig. A-1 CONSORT schematic trial design. *Optional study modules.

Additional file 3: SPIRIT checklist.

## Data Availability

Beyond the registration in the German Clinical Trial Register (Identifier: DRKS00024898), CogTrAiL-RBD is registered in the Open Science Framework (OSF, https://osf.io/). The OSF will be used as a platform to make our data and corresponding material available. Following the publication of corresponding manuscripts, we aim to share the anonymized individual participant data (IPD) according to the FAIR principle—findable, accessible, interoperable, reusable. All data sharing will only be undertaken in accordance to legal requirements. A model consent form is displayed in Appendix B-2.

## Abbreviations

CogTrAiL-RBD: Cognitive Training and a Healthy, Active Lifestyle Program for Patients with isolated REM Sleep Behavior Disorder
DLB: Dementia with Lewy bodies
HC: Healthy control individuals
ICC: Intraclass correlation
iRBD: Isolated REM (rapid eye movement) sleep behavior disorder
IPAQ: International Physical Activities Questionnaire
LAQ: Lifestyle Activities Questionnaire
MDS-UPDRS-III: Movement Disorder Society Unified Parkinson’s Disease Rating Scale Part III
MEDAS: Mediterranean Diet Adherence Screener (MEDAS)
MRI: Magnetic resonance imaging
MSA: Multiple system atrophy
OSF: Open Science Framework
PD: Parkinson’s disease
PDD: Parkinson’s disease dementia
PR: Percentage ranks
RBD: REM (rapid eye movement) sleep behavior disorder
SPIRIT: Standard Protocol Items: Recommendations for Interventional Trials
TE: Echo time
TR: Repetition time
