# Supplementary material for: Cognitive training and promoting a healthy lifestyle for individuals with isolated REM sleep behavior disorder: study protocol of the delayed-start randomized controlled trial CogTrAiL-RBD
Source: Trials. 2024 Jun 28;25:428. doi: 10.1186/s13063-024-08265-9 (PMC11214208; doi:10.1186/s13063-024-08265-9)
Supplement: Supplementary file 1 — Additional file 1: Appendix Figure A-1 and Appendix B-2. [file 13063_2024_8265_MOESM1_ESM.pdf]

Cognitive training and a healthy lifestyle program

for individuals with isolated REM sleep behavior disorder:

study protocol of the delayed-start randomized controlled trial CogTrAiL-RBD

Appendix Figure A-1

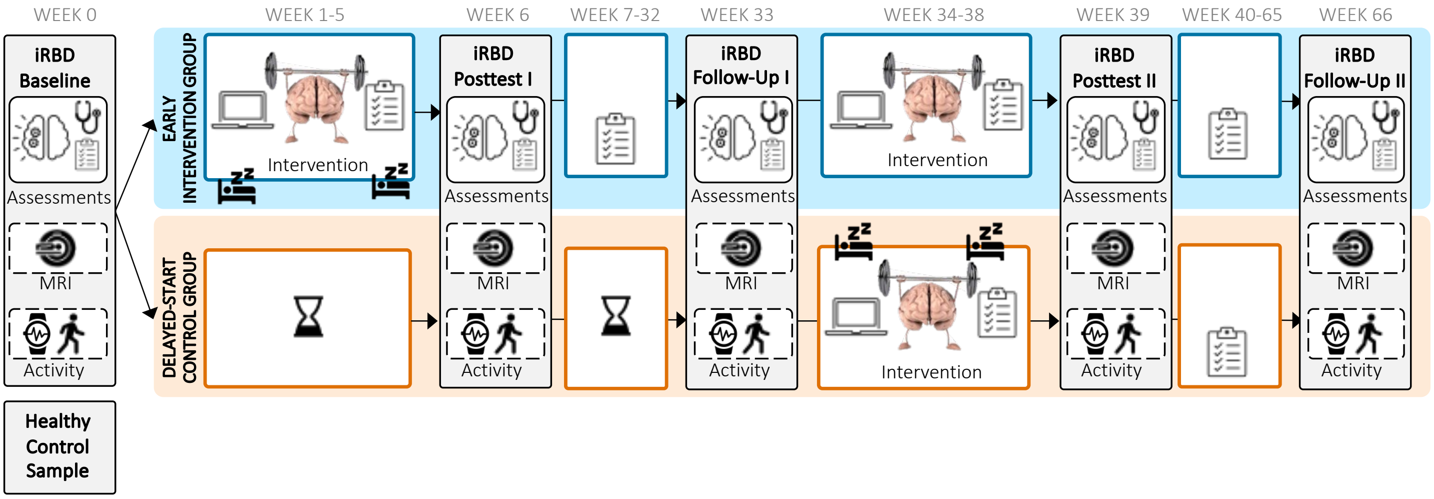

Fig. A-1 Study Design of CogTrAiL-RBD

iRBD, isolated REM sleep behavior disorder; MRI, magnetic resonance imaging. All participants with iRBD will undergo comprehensive clinical and neuropsychological assessments ("Assessments") at the t0 visit and 6 weeks post allocation (Posttest I, t1, week 6). A follow-up assessment (Follow-Up I, t2, week 33) will be conducted after 6 months. Subsequently, the second intervention phase for both the early intervention group and the delayed-start control group begins, followed by another clinical and neuropsychological assessment (Posttest II, t3, week 39). Finally, a follow-up assessment (Follow-Up II, t4, week 66) will take place after additional 6 months. There are three optional study modules for individuals with iRBD that accompany the mandatory clinical and neuropsychological assessments: (i) the MRI module, (ii) the accelerometry module ("Activity"); and (iii) the polysomnography module (sleep symbol). A healthy, age-matched HC group will be examined at the first assessment time point, participating in the comprehensive clinical and neuropsychological assessments, the MRI module and the accelerometry module.

Appendix B-2 Model consent form

UNIKLINIK KÖLN

Medizinische Psychologie | Neuropsychologie & Gender Studies

AG Prodromalstadien und Schlaf bei Bewegungsstörungen der Klinik und Poliklinik für Neurologie

Kognitives Training & Programm zu einem aktiven, gesunden Lebensstil für Patient*innen mit idiopathischer REM-Schlaf-Verhaltensstörung

CogTrAiL-RBD

*Ihre Ansprechpartner*innen*

Medizinische Psychologie | Neuropsychologie und Gender Studies
und
AG Prodromalstadien und Schlaf bei Bewegungsstörungen
der Klinik und Poliklinik für Neurologie
Medizinische Fakultät und Uniklinik Köln, Universität zu Köln
Kerpenerstr. 62
D-50937 Köln

Univ.-Prof. Dr. rer. nat. Elke Kalbe
+49 221 478-6669 (Sekretariat); elke.kalbe@uk-koeln.de

Dr. phil. Anja Ophey, M.Sc., Psychologin
+49 221 478-32976; anja.ophey@uk-koeln.de

Priv. Doz. Dr. med. Michael Sommerauer
+49 221 478-98566; michael.sommerauer@uk-koeln.de

Einwilligungserklärung

CogTrAiL-RBD [Allgemein]

o Herr o Frau

Name: ____

Vorname: ____

Geburtsdatum: ____

- Ich habe die Information erhalten und wurde über Wesen, Bedeutung, Tragweite und Risiken des geplanten Vorhabens informiert. Mir wurde ausreichend Gelegenheit gegeben, alle offenen Fragen zu klären. Ich habe jederzeit das Recht, weitere Informationen zur Studie zu erfragen.
- Ich erkläre mich freiwillig bereit, an den folgenden Modulen der Studie **„CogTrAiL-RBD"** teilzunehmen:
  - Neuropsychologische und motorische Untersuchungen an der Uniklinik Köln, Ausfüllen von Fragebögen, Intervention bestehend aus einem digitalen kognitiven Training + Modul zu einem aktiven, gesunden Lebensstil sowie…

  ☐ MRT-Untersuchungen am Forschungszentrum Jülich

  ☐ Schlafuntersuchungen

  ☐ Aktivitätsmessungen im Alltag
- Ich habe jederzeit das Recht, ohne Angabe von Gründen von der Studie zurückzutreten, ohne dass für mich Nachteile in der medizinischen Behandlung daraus entstehen.

Einwilligungserklärung zum Datenschutz:
Bei dieser wissenschaftlichen Studie werden personenbezogene Daten und medizinische Befunde über Sie erhoben. Die Speicherung, Weitergabe und Auswertung dieser Daten erfolgt gemäß gesetzlichen Bestimmungen und setzt vor Teilnahme an der Studie die folgende freiwillige Einwilligung voraus:

1. Ich erkläre mich damit einverstanden, dass im Rahmen dieser Studie erhobene Daten auf Fragebögen und elektronischen Datenträgern in pseudonymisierter Form (ohne Namensnennung) aufgezeichnet und an die Auftraggebenden der Studie/ die Studienleitung / die Gesamtverantwortlichen für die Studie weitergegeben werden.
   ☐ JA ☐ NEIN
2. Ich bin damit einverstanden, dass die neurologische Untersuchung zur Beurteilung der Motorik auf Video aufgezeichnet und gespeichert wird. Die Videoaufzeichnungen werden nur für die Beurteilung der Motorik verwendet, die Auswertung der motorischen Untersuchung wird pseudonymisiert gespeichert. Nach Auswertung erfolgt eine direkte Löschung der Videos.
   ☐ JA ☐ NEIN
3. Ich erkläre mich damit einverstanden, dass die während des kognitiven Trainings mit NEUROvitalis Digital am Computer erhobenen und gespeicherten Daten von der HelferApp GmbH an die obengenannten Verantwortlichen weitergegeben werden.
   ☐ JA ☐ NEIN
4. Ich erkläre mich damit einverstanden, dass meine Kontaktdaten gespeichert werden, damit mir Informationen über weitere Studien übermittelt werden können.
   ☐ JA ☐ NEIN
5. Außerdem erkläre ich mich damit einverstanden, dass die im Rahmen dieser Studie erhobenen Daten pseudonymisiert für Folgeanalysen aus dem Bereich der medizinischen Psychologie und der kognitiven Neurowissenschaften und für zukünftige wissenschaftliche Studien der Kliniken für Neurologie und Psychiatrie der Uniklinik Köln sowie des Instituts für Neurowissenschaften und Medizin (INM) des Forschungszentrum Jülich zur Verfügung gestellt werden können. Die Weiterverarbeitung Ihrer Daten erfolgt ausschließlich unter dem Zweck der wissenschaftlichen Forschung durch Stellen, die ein geeignetes Datenschutzkonzept vorweisen können.
   ☐ JA ☐ NEIN
6. Ich gebe mein Einverständnis, dass die im Rahmen dieser Studie erhobenen Daten pseudonymisiert mit Mitarbeitenden anderer Forschungsgruppen universitärer und außeruniversitärer Forschungseinrichtungen für eine vertiefte Datenanalyse nicht-kommerzieller Forschungsprojekte geteilt werden dürfen (z.B. Daten der Aktivitätssensoren und MRT-Daten, wenn zutreffend).
   ☐ JA ☐ NEIN
7. Außerdem erkläre ich mich damit einverstanden, dass ein*e autorisierte*r und zur Verschwiegenheit verpflichtete*r Beauftragte*r der Auftraggebenden/der Studienleitung in meine beim Studienarzt*bei der Studienärztin vorhandenen personenbezogenen Daten Einsicht nehmen kann, soweit dies für die Überprüfung der Studie notwendig ist. Für diese Maßnahmen entbinde ich den Studienarzt*die Studienärztin von der ärztlichen Schweigepflicht.
   ☐ JA ☐ NEIN

8. Für eventuell anfallende Fahrt- und/oder Parkkosten, die im Rahmen meiner Studienteilnahme anfallen, wurde mir eine Aufwandsentschädigung in Höhe von 10 Euro pro Testtag in der Uniklinik Köln, d.h. maximal 50 Euro (plus maximal 20 Euro pro Testtag am Forschungszentrum Jülich, d.h. plus 100 Euro, insgesamt also 150 Euro, wenn ich zusätzlich am Modul Bildgebung am Forschungszentrum Jülich teilnehme) per Überweisung auf mein Konto angeboten. Mir wurde mitgeteilt, dass die Auszahlung dieses Betrags ausschließlich per Überweisung nach Abschluss des letzten Untersuchungstermins erfolgen kann. Hiermit erkläre ich, dass…

   ☐ ich eine Aufwandsentschädigung für die entstandenen Kosten (mit einer maximalen Grenze von 10 Euro pro wahrgenommenem Testtag an der Uniklinik Köln bzw. 20 Euro pro wahrgenommenem Testtag am Forschungszentrum Jülich) per Überweisung auf mein Konto erhalten möchte und meine Kontodaten ausschließlich für diesen Zweck hier mitteile:

   IBAN: |_||_||_||_||_||_||_||_||_||_||_||_||_||_||_||_||_||_||_||_||_||_|

   BIC: |_||_||_||_||_||_||_||_|

   ☐ ich auf eine Aufwandsentschädigung verzichte.
9. Ich erkläre mich damit einverstanden, dass meine Daten nach Beendigung oder Abbruch der Studie bis zu 10 Jahre nach Ende der Studie gemäß wissenschaftlichem Standard aufbewahrt werden. Danach werden meine personenbezogenen Daten gelöscht oder anonymisiert.
   ☐ JA ☐ NEIN
10. Ich bin darüber aufgeklärt worden, dass ich jederzeit die Teilnahme an der Studie beenden kann. In diesem Falle werden bereits erhobene Daten gelöscht oder vollständig anonymisiert.
    ☐ JA ☐ NEIN
11. Ich habe die Informationen zum Datenschutz zur Kenntnis genommen und willige in die Datenverarbeitung ein.
    ☐ JA ☐ NEIN

Ich habe die vollständige Proband*inneninformation zur Studie, die Versicherungsbestätigung und Versicherungsbedingungen, sowie ein unterschriebenes Exemplar dieser Einwilligungserklärung erhalten.

______________________	______________________	______________________
Vor- und Nachname des Studienteilnehmers*der Studienteilnehmerin	Ort und Datum (persönlich auszufüllen)	Unterschrift des Studienteilnehmers*der Studienteilnehmerin (in Druckbuchstaben)

Ich habe das Aufklärungsgespräch geführt und die Einwilligung des Studienteilnehmers*der Studienteilnehmerin eingeholt.

______________________	______________________	______________________
Vor- und Nachname des*r Studienärztin/Studienarztes (in Druckbuchstaben)	Ort und Datum (persönlich auszufüllen)	Unterschrift des*r Studienärztin/Studienarztes
