# Supplementary material for: Cognitive training and promoting a healthy lifestyle for individuals with isolated REM sleep behavior disorder: study protocol of the delayed-start randomized controlled trial CogTrAiL-RBD
Source: Trials. 2024 Jun 28;25:428. doi: 10.1186/s13063-024-08265-9 (PMC11214208; doi:10.1186/s13063-024-08265-9)
Supplement: Supplementary file 2 — Additional file 2: Fig. A-1 CONSORT schematic trial design. *Optional study modules. [file 13063_2024_8265_MOESM2_ESM.docx]

**Fig. A-1** CONSORT schematic trial design

|  | **STUDY PERIOD** | | | | | |
| --- | --- | --- | --- | --- | --- | --- |
|  | **Enrolment** | **Allocation** | **Post-allocation** | | | **Close-out** |
| **TIMEPOINT** | **-t1** | **t0** | **t1** | **t2** | **t3** | **t4** |
| **ENROLMENT** |  |  |  |  |  |  |
| Eligibility screen | X | X |  |  |  |  |
| Informed consent | X |  |  |  |  |  |
| Allocation |  | X |  |  |  |  |
| **INTERVENTIONS** |  |  |  |  |  |  |
| Early intervention group |  | X---------------X | | X---------------X | |  |
| Delayed-start group |  |  | | X---------------X | |  |
| **ASSESSMENTS** |  |  |  |  |  |  |
| Neuropsychology: Cognitive testing |  | X | X | X | X | X |
| Neuropsychology: Questionnaires |  | X | X | X | X | X |
| Motor assessments |  | X | X | X | X | X |
| Accelerometry |  | X* | X* | X* | X* | X* |
| Magnetic Resonance Imaging |  | X* | X* | X* | X* | X* |
| Polysomnography |  | X*---------------X* | | X*---------------X* | |  |

* optional study modules
